# mSphere of Influence: the Power of Yeast Genetics Still Going Strong!

**DOI:** 10.1128/mSphere.00647-19

**Published:** 2019-10-02

**Authors:** Felipe H. Santiago-Tirado

**Affiliations:** a Department of Biological Sciences, University of Notre Dame, Notre Dame, Indiana, USA

**Keywords:** *Cryptococcus neoformans*, budding yeast, yeast genetics

## Abstract

Felipe Santiago-Tirado studies the cell biology of cryptococcal infections. In this mSphere of Influence article, he reflects on how the papers “Systematic Genetic Analysis of Virulence in the Human Fungal Pathogen Cryptococcus neoformans” (https://doi.org/10.1016/j.cell.2008.07.046) and “Unraveling the Biology of a Fungal Meningitis Pathogen Using Chemical Genetics” (https://doi.org/10.1016/j.cell.2014.10.044) by the Noble and Madhani groups influenced his thinking by showcasing the various modern applications of yeast genetics in an organism where genetic manipulation was difficult.

## Abstract (precis)

Felipe Santiago-Tirado studies the cell biology of cryptococcal infections.

## COMMENTARY

Despite referring to a type of cellular division that many organisms use, the term “budding yeast” is universally known to refer to the yeast Saccharomyces cerevisiae. This yeast is one of the most well-studied, genetically tractable organisms, which drove the start of the modern molecular biology era and was instrumental in dissecting fundamental biology that was relevant to all eukaryotes, including humans. Revolutionary techniques and tools were developed in this organism, resulting in many advances in the fields of genomics, proteomics, physiology, and microscopy that have been recognized by several prestigious awards, including 7 Nobel Prizes (1). Thanks to this yeast, I obtained a PhD and became an expert in the fields of membrane trafficking, polarity establishment, and, more generally, yeast genetics. This is the point when I, very enthusiastically, decided to go and work with a yeast with even more human relevance, another budding yeast called Cryptococcus neoformans. Unlike the friendly budding yeast, C. neoformans is one of the leading causes of invasive fungal infections, responsible for ∼200,000 deaths yearly, and toward which there is a limited number of inadequate treatments available (2). Little did I know, when I changed fields, that the two budding yeasts, although similar in size and morphology, could not be more different. To start, C. neoformans is not a genetically tractable organism, it is difficult to transform, it seems to actively avoid homologous recombination, and the genetic structure is most similar to that of humans rather than to other yeasts. So, how would I ever be able to apply all of those powerful techniques I learned with S. cerevisiae? Reading the paper “Systematic Genetic Analysis of Virulence in the Human Fungal Pathogen Cryptococcus neoformans” (3) gave me hope, but the subsequent paper, “Unraveling the Biology of a Fungal Meningitis Pathogen Using Chemical Genetics” (4), clearly demonstrated that it was possible. The power of yeast genetics could still be applied in this field, opening a whole new world of ideas and approaches.

In “Systematic Genetic Analysis of Virulence in the Human Fungal Pathogen Cryptococcus neoformans” (3), the authors built the first genomic resource in the field, a partial deletion collection that still is ongoing. They used this collection to study the classical virulence factors of the fungus, but also devised a method to study virulence *in vivo* in a way that allows high throughput but minimizes the number of animals needed. This paper not only provided a plethora of useful data, but also made available to the community an incredible useful resource. In the follow-up paper, “Unraveling the Biology of a Fungal Meningitis Pathogen Using Chemical Genetics” (4), using a slightly larger deletion collection, the authors performed a systematic chemical-genetic interaction screen, another genomic assay first developed in S. cerevisiae (5). This type of assay is based on the observation that the effects of a chemical compound on cell growth (or another easily measured phenotype) are dependent on particular genes. By correlating and grouping similar profiles (i.e., two drugs affecting similarly a group of mutants, or if two mutants show the same or similar drug interactions), they were able to identify novel pathogenicity genes, infer compound mode of action, and develop an algorithm that predicts antifungal synergies. These two papers are clear proof that powerful systematic genome-wide approaches, based on original yeast genetic techniques, can still be applied in C. neoformans, allowing us fungal researchers to discover unique cryptococcal biology faster and more efficiently.

Since the publication of that first partial deletion collection, several advances in the cryptococcal field have occurred that will certainly have a positive impact in the research. CRISPR has been adapted and is now possible in this organism (6). The deletion collection is almost complete and is now expanding into other types of genome-wide collections (such as one where each protein-coding gene is epitope tagged [NIH grant 5R01AI100272]). These new resources, together with my knowledge of the “classical” yeast genetics, have enabled me to develop strategies that otherwise I would not have ever consider. I can go back to my days working with the friendly budding yeast and think “What would I do if I wanted to tackle this question?” and then go and try it in C. neoformans. Moreover, these papers were among the first to apply classical yeast genetics to a fungal pathogen on a large scale, hence their impacts cross boundaries and have influence other fungal researchers, such as those in the *Candida* and *Aspergillus* fields (7, 8). Using these resources together with recent advances such as CRISPR or multiplexed whole-genome sequencing, I believe that we will be able to move the cryptococcal field forward almost at the same rate that budding yeast, the friendly one, did in the past.
